# Exhaled particles and small airways

**DOI:** 10.1186/s12931-019-0970-9

**Published:** 2019-01-11

**Authors:** B. Bake, P. Larsson, G. Ljungkvist, E. Ljungström, A-C Olin

**Affiliations:** 1 Unit of Respiratory Medicine and Allergy, Department of Internal Medicine, Institute of Medicine, Sahlgrenska Academy, University of Gothenburg, Gothenburg, Sweden; 2 Unit of Occupational and Environmental Medicine, Department of Public Health and Community Medicine, Institute of Medicine, Sahlgrenska Academy, University of Gothenburg, Gothenburg, Sweden; 3 Atmospheric Science, Department of Chemistry and Molecular Biology, University of Gothenburg, Gothenburg, Sweden

**Keywords:** Exhaled particles, Small airways, Airway closure, Airway opening, Surfactant, Proteomics, SP-A, Albumin, DPPC, POPC

## Abstract

**Background:**

Originally, studies on exhaled droplets explored properties of airborne transmission of infectious diseases. More recently, the interest focuses on properties of exhaled droplets as biomarkers, enabled by the development of technical equipment and methods for chemical analysis. Because exhaled droplets contain nonvolatile substances, particles is the physical designation. This review aims to outline the development in the area of exhaled particles, particularly regarding biomarkers and the connection with small airways, i e airways with an internal diameter < 2 mm.

**Main body:**

Generation mechanisms, sites of origin, number concentrations of exhaled particles and the content of nonvolatile substances are studied. Exhaled particles range in diameter from 0.01 and 1000 μm depending on generation mechanism and site of origin. Airway reopening is one scientifically substantiated particle generation mechanism. During deep expirations, small airways close and the reopening process produces minute particles. When exhaled, these particles have a diameter of < 4 μm. A size discriminating sampling of particles < 4 μm and determination of the size distribution, allows exhaled particle mass to be estimated. The median mass is represented by particles in the size range of 0.7 to 1.0 μm. Half an hour of repeated deep expirations result in samples in the order of nanogram to microgram. The source of these samples is the respiratory tract ling fluid of small airways and consists of lipids and proteins, similarly to surfactant. Early clinical studies of e g chronic obstructive pulmonary disease and asthma, reported altered particle formation and particle composition.

**Conclusion:**

The physical properties and content of exhaled particles generated by the airway reopening mechanism offers an exciting noninvasive way to obtain samples from the respiratory tract lining fluid of small airways. The biomarker potential is only at the beginning to be explored.

**Electronic supplementary material:**

The online version of this article (10.1186/s12931-019-0970-9) contains supplementary material, which is available to authorized users.

## Background

Exhaled air is an aerosol containing endogenously generated droplets. These droplets contain water and nonvolatile material, and “particles” is therefore the physical designation, even though they are liquid droplets. Studies of exhaled particles originally aimed to understand the transmission of airborne infections. More recently, however, interest has extended to include a search for biomarkers of pathology in the airways. The close proximity to pathological processes in the airways makes exhaled particles an attractive option for clinical investigation. Knowledge of the site of origin and mechanisms of generation of exhaled particles constitutes an important basis for exploring associated biomarkers.

Along with exhaled particles, volatile- and semi-volatile substances may carry important biomarkers such as exhaled nitric oxide [1, 2], e g regarding the effect of the injury on small airways due to the mechanical stress following cyclic opening and closure among patients with chronic obstructive pulmonary disease (COPD) [3]. Exhaled Breath Condensate and certain physiological methods also contribute to the diagnosis of small airway disease [4–6]. The present review, however, focuses on endogenously produced exhaled particles originating in the respiratory tract lining fluid (RTLF) along the airways including the pharynx and mouth. It is limited to studies presenting the count number and size distribution of exhaled particles, therefore a wide range of important studies on small airways have been omitted. We have highlighted key studies reporting the increasing appreciation of the origin and characteristics of exhaled particles. We also present available information on the use of exhaled particles from small airways as biomarkers.

### Particle size

It is very difficult to directly determine size of small droplets (diameter < 10 μm) floating in air. In practice, however, a measured property that depends on particle size is commonly used to indirectly estimate the size. Additional file 1, “Technical and methodological considerations,” outlines the various sizing methods employed in the studies reported here. Presumably, particles generated in situ and then exhaled are liquid spheres. Aqueous droplets will equilibrate with the water vapor in the surrounding air. It follows that their size depends on the surrounding air temperature and humidity as well as the particles’ composition. Equilibration is a rapid process (< 1 s) for small droplets, but may be confounded by the presence of a surfactant layer covering the droplet’s surface slowing evaporation or condensation.

### Collection of exhaled particles

Chemical analysis requires sampling exhaled particles. The design of the sampling equipment will inevitably affect the size range of the collected sample. For examples, long tubing, parts not at roughly 35 °C, and sharp turns will contribute to losses of particles, particularly those that are relatively large.

The impactor is a sampling device that allows the collection of a size discriminated samples from an aerosol. Details are presented in Additional file 1.

### Chemical analysis of collected exhaled particles

The great challenge analytically is the extremely low amounts of collected analytes, in the range of picogram (pg) per liter of exhaled air. Electron microscope and X-ray dispersive analysis or surface mass spectrometry, e.g. time-of-flight secondary ion mass spectrometry (TOF-SIMS) can analyze the deposited particles directly [7, 8]. Exhaled particles collected by impaction need to be appropriately desorbed. For proteins, immunological methods have dominated so far, but mass spectrometric methods have emerged for proteins as well as for lipids [9, 10]. Proteomics analysis has been able to quantify over 200 proteins by combining DNA-markers with PCR amplification of small amounts of particles (in the order of hundred ng) [11].

### Historical perspectives

More than 70 years ago Duguid [12] aimed to assess the mechanisms of airborne transmission of infection from the mouth and throat. Five participants performed different breathing maneuvers, including normal mouth breathing, counting softly and loudly from 1 to 100, and performing various cough maneuvers. Immediately before these maneuvers, he had applied bacteria to the mucous membranes of the throat and nose. In a separate session, he applied a dye to the surfaces of the mouth, front teeth, lips, and tip of the tongue. Exhaled particles ended up either on a bacterial growth medium or on a glass slide for particle counting using a microscope.

Results from normal mouth breathing revealed no exhaled droplets > 20 μm in any of 15 one-minute tests using directly exposed culture plates. Counting softly resulted in 63 (range 0–160) stain-containing droplets between 1 and 100 μm; counting loudly resulted in 4 to 14 times higher counts. Cough results were dependent on the cough performance; “tongue-teeth cough” gave average counts of 8200, presumably depending among other things on the location and concentration of the dye*.* The particle counts were many times higher than the colony counts, presumably because many small particles did not contain bacteria.

*Comment: Studies were on particles generated in the upper airways only. There was no information on the particles < 20* μm *exhaled during normal mouth breathing. Particles > 20 μm were indeed exhaled during all other breathing activities, except for normal breathing.*

About 20 years later Loudon and Roberts [13] aimed to determine the numbers and sizes of exhaled particles using a sampling technique that allowed comparisons of the frequency distribution of all particles > 1 μm. Three participants in two experiments performed a series of 15 coughs into a box and in two other experiments counted loudly from 1 to 100 into the box. Before each experiment, the participant swabbed the inside of his mouth with dye. After each experiment, the box was closed and particles settled on paper slips over 30 min. Settled particles were counted, as were the remaining airborne particles, which were deposited on a Millipore filter in the exit port of the box.

The results from the three individuals showed that the median diameter of particles generated during talking and coughing were 81 μm and 26 μm respectively. Six percent of the particles generated during talking remained airborne after 30 min versus 49% of the particles generated during coughing, indicating the importance of coughing in the transmission of bacterial infections. The number of particles produced by coughing varied broadly. The authors discussed several potentially important causes of the large variability: coughing is difficult to standardize; particle formation depends on a number of factors including the amount of secretion and its location in the mouth and the placement and movement of the lips, tongue, and teeth.

*Comments: Studies were limited to particles generated in the mouth and there is no information on exhaled particles during normal breathing.*

In 1997 Papineni and Rosenthal [14] presented results on droplets in exhaled breath obtained by two methods: (a) a real-time analysis by an optical particle counter (OPC) and (b) analysis of dried droplet residues by electron microscopy. The mouth was without dye, and consequently the site of origin of the exhaled particles was not necessarily the mouth region. The OPC and associated software presented particle sizes in six channels between 0.3 and 2.5 μm. Nose breathing, mouth breathing, coughing, and talking were studied in five healthy participants using the OPC and electron microscopic analysis of the mouth breathing particles was conducted with three of the participants.

Results according to the OPC method showed that mouth breathing resulted in 12.5 particles/L for diameters < 1 μm and 1.9 particles/L for diameters > 1 μm. Coughing resulted in 83.2 particles/L for diameters < 1 μm and 13.4 particles/L for diameters > 1 μm.

The results from electron microscopy showed that the size distribution was more heavily weighted towards larger particles: original droplet sizes > 1 μm constituted 64% and the largest particle was 7.6 μm. As the droplet size estimates from the electron microscope were considered to be unaffected by evaporation, the OPC method may have underestimated the original droplet size through evaporation and/or losses of large particles in the funnel.

*Comments: Normal breathing does indeed exhale submicron as well as larger particles*. *The site of origin and mechanisms of generation are still unknown; however, X-ray dispersive analysis of the residue of one particle revealed contents of potassium, calcium, and chloride, consistent with RTLF origin.*

In 2004 Edwards et al. [15] investigated the ability to transiently diminish the number of exhaled particles by administering nebulized aerosols to human participants. Particles were measured by an OPC providing counts in six bins between 0.09 – > 0.5 μm. Eleven healthy participants were investigated on three visits separated by at least a week in a crossover placebo-controlled design. An aerosol was inhaled on the two first visits, either isotonic saline with a surface tension of 72 dyne/cm or a surfactant simulant consisting of 1,2-dipalmitoyl-*sn*-glycero-3-phosphocholine (DPPC) or 1-palmitoyl-2-oleoyl-*sn*-glycero-3-phosphoglycerol with a surface tension of 42 dyne/cm. No aerosol treatment was conducted on the third visit. Participants wore nose clamps and breathed large tidal volumes of close to 1 L, inhaling particle-free air. Exhaled particles were measured for 2 min immediately before and 5 min, 30 min, 1 h, 2 h, and 6 h after inhalation.

The results showed that without aerosol treatment, particle number concentration varied among participants between 1 and 10,000/L exhaled air and also varied considerably within participants between the six measurements during each visit. The authors subdivided the results into high (*n* = 6) and low (*n* = 5) particle producers and found that saline delivery resulted in a statistically significant drop of particle emission among high producers and a tendency to increase emission among low producers. Administration of the surfactant simulant amplified particle emission by a factor of about five! No effects on particle size distribution were observed following administration of the saline or the surfactant simulant and the predominant particle size was 0.15–0.2 μm. The diminished particle emission after saline administration among high particle producers was explained by a presumed shift towards large particles outside the OPC’s range resulting in a substantial fraction of particles to deposit in the airways.

Experiments performed on a cough machine consisting of a model trachea lined on the bottom with a mucus simulant showed that saline or surfactant administration resulted in a dramatic shift in the size distribution 30–60 min after administration from 0.2 μm to about 30 μm.

*Comments: Subdivision into high and low particle producers is probably misleading. Recent studies showed that exhaled particles are distributed approximately log normally with no sign of two size modes* [16, 17]*. Considering all participants, there was no significant reduction of exhaled particles within the size range of the OPC. Administration of a surfactant simulant to the participants substantially increased particle emission, indicating that surface tension is important. The surfactant simulant with a surface tension of about 42 dyn/cm may in fact increase the surface tension of small airways, particularly at low lung volumes when the surface tension is normally close to zero. Increased surface tension increases particle production* [18, 19]*. Conclusions from the cough machine results are relevant to coughing and forced exhalation, but not to human tidal breathing. The cough machine generates particles by the burst of air destabilizing the mucus/air interface by shear forces to form submicron droplets.*

Watanabe et al. [20] studied in vitro effects, particularly of isotonic sodium chloride, on the propensity of RTLF to form small droplets of various aerosolized formulations and widely varying surface tensions and viscoelastic properties. The main experiments were performed on the cough machine used by Edwards et al. [15], which was altered by reducing the applied air pressure from about 126 kPa to about 21 kPa to simulate a less violent breathing maneuver. The model measured particle production caused by simulated breathing over the mucus mimetic trachea after the various aerosolized formulations were applied. Particle production was assessed by an OPC covering particle sizes between 0.09 and > 0.5 μm.

The results showed that application of salt solutions with and without other additives increase the surface viscoelasticity relative to the mucus mimetic alone and that gelation of the free surface of the RTLF mimetic resulted in a significant diminution of aerosol particle generation. Experiments on calf lungs confirmed that the charge-mediated gelation near the surface of the RTLF mimetic was reversible.

*Comments: The simulated breathing maneuver corresponds to a rather forceful expiration. Under these circumstances, the trachea model reveals a new mechanism: the RTLF/air interface may be stabilized by gelation of the mucus by salt water, making RTLF less prone to disintegrate into very small particles.*

**Fig. 1:**
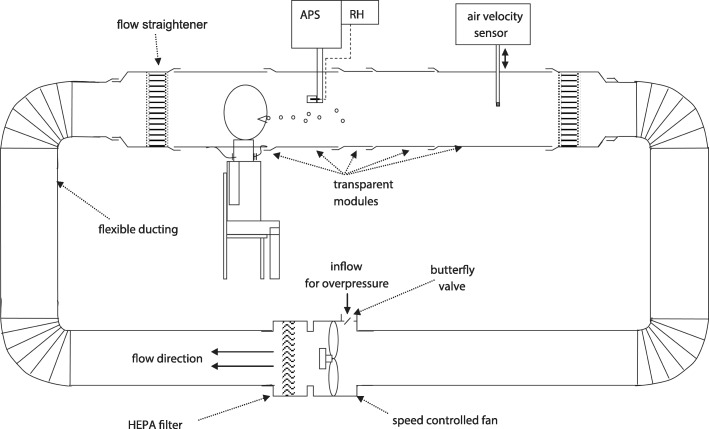
The Expiratory Droplet Investigation System setup used by Morawska et al. [21]. Test participants exhaled into a particle- free wind tunnel. A fan maintained at approximately 0.1 m/s controls the wind tunnel airflow. The airflow transports exhaled particles downstream to the aerodynamic particle sizer (APS) where the particles are measured. A relative humidity (RH) probe monitors the humidity. Reprinted from the original article by permission

In 2009 Morawska et al. [21] studied exhaled particle concentrations and size distributions at the mouth using a new investigation system. The system was essentially a small wind tunnel, into which participants could place their heads (see Fig. 1). An aerodynamic particle sizer (APS) measured particles with diameters ranging mainly from 0.5 to 20 μm. A sample of 15 healthy participants aged ≤35 years performed the following breathing exercises at a rate and depth which felt most natural: (a) in through the nose and out through the mouth, (b) in through the nose and out through the nose, (c) whispering “aah”, (d) voicing “aah”, (e) whispering counting, (f) voicing counting, and (g) coughing. Samples were counted for 2 min and repeated three times with 20 min rests between counts. The statistical analysis applied a so-called mixture model, assuming that observed results were a superposition of log normal distributions representing the various breathing maneuvers.

Table 1 (extracted from their Fig. 5) presents results for concentrations of exhaled particles with diameters from 0.5 to 20 μm.

**Table 1:** Exhaled particles during various breathing maneuvers

Breathing maneuver	Exhaled particle number concentration (n/L)
in through the nose and out through the mouth	98
whispering “aah”	672
voiced “aah”	1088
whispering counting	100
voiced counting	130
cough	678

Voiced activities resulted in higher particle concentrations than whispered, indicating that the vibrating vocal cords during vocalization produce exhaled particles. Whispered counting produced similar concentrations as normal mouth breathing, indicating that gentle movements of the lips and tongue generate very few exhaled particles in the size range of 0.5–20 μm. Whispering “aah” generated as many exhaled particles as coughing, indicating that high air velocity passing an almost closed epiglottis is an effective particle generating mechanism. The particle size at maximum concentration was about 0.8 μm and there were few particles > 10 μm. The mixture model provided a good fit with four modes (A, B, C, D) for all breathing activities: A is associated with normal breathing; B, C, and D are associated with vocalization and epiglottis adduction. Count median diameters were ≤ 0.8 μm, 1.8 μm, 3.5 μm, and 5.5 μm respectively.

*Comments: In agreement with Papineni and Rosenthal* [14] *the results show that normal mouth breathing generates particles but no generation mechanism is suggested. During vocalization, however, vibrating vocal cords and air passage through adducted epiglottis almost certainly produces exhaled particles.*

Chao et al. [17] measured the droplet size distribution in close proximity to the mouth opening during coughing and speaking. A sample of 11 healthy volunteers under 30 years of age were asked to count loudly and slowly 10 times from 1 to 100. After a break, they coughed 50 times with lips closed before each cough.

The size measurements at 10 mm from the mouth were regarded to be unaffected by evaporation and condensation and to be representative of the “original” size profile. The particles were distributed in 16 size classes with mean values ranging from 3 to 1500 μm. The size class with the highest number count was 6 μm for both speaking and coughing, and the geometric mean diameter was 16.0 μm for speaking and 13.5 μm for coughing. The size distribution during both speaking and coughing was highly skewed with small numbers of large particles.

*Comment: This study is the first to measure the size interval from about 2* μm *to 2000 μm with the same measuring system and with an experimental set-up optimized to measure particles unaffected by evaporation/condensation during speaking and coughing. The counts of particles in the largest size classes were very low but represented almost all the volume and mass. It is worth bearing in mind that the mass of one particle with a diameter of 150 μm corresponds to almost 6.6 million particles with a diameter of 0.8 μm assuming similar density and spherical shapes.*

### Airway reopening hypothesis

At about the same time as the study discussed above, several independent groups dealt with the notion that one important mechanism for particle generation is the reopening of closed airways [18, 22–24] – as previously posited by Edwards et al. [15]. The fact that small peripheral airways normally close following a deep expiration was originally shown by Milic-Emili and coworkers in 1966–1968 [25–27] and elegantly confirmed by Burger and Macklem [28] and Engel et al. [29]. In upright position, the apical parts of the lungs are more expanded than basal parts due to the weight of the lungs. During an expiration to low lung volumes, the basal airways collapse with the airway walls pasted together by RTLF and reopen again on inspiration. There is a simple single-breath test to determine the volume at which extensive airway closure (the closing volume) begins [30]. To the best of our knowledge, the precise location of airway closure along the airway tree is not known in humans but is generally considered to be in the small airways. In dogs, airway closure appears to take place in airways with an internal diameter of 0.4–0.6 mm [31]. Some airways may close at higher lung volumes than indicated by the closing volume [32], and massive airway closure may occur during tidal breathing [33] at low lung volumes (low functional residual capacity) as in people who are obese or whose closing volumes are increased by a disease such as COPD [32]. Then there is a risk of mechanical injury of the small airways due to the cyclic closing and reopening [33].

Johnson and Morawska [23] using the same equipment as Morawska et al. [21], including the Aerodynamic Particle sizer (APS) to determine exhaled particles in the diameter range of 0.5–20 μm. Seventeen participants between 19 and 60 years of age took part. Four different breathing activities were performed and were repeated for 2-min periods:

1. Inspiring a normal breath volume via the nose and exhaling via the mouth.
2. Inspiring a normal breath volume via the mouth over a 3-s period, followed immediately by a 1-s full deep exhalation.
3. Rapid inspiration of a normal breath volume via the mouth, followed by holding the breath for 2, 3, 5, or 10 s and full deep exhalation over 3-s;
4. Inspiring a normal breath volume via the mouth over a 3-s period, followed immediately by a 3-s full deep exhalation.

The results show that deep exhalations increased the exhaled particle concentrations. Furthermore, breath holding at mid lung volume was found to reduce the exhaled particle concentrations proportional to the duration of breath holding and to cause a shift towards smaller particles. The breath-holding results fit with the predicted effects of gravitational settling in an alveolus considering that droplet size is about two times larger in the alveolus than when measured because of shrinkage during exposure to ambient humidity. The humidity correction was later experimentally confirmed by Holmgren et al. [34]. A rather weak positive correlation with age was reported but one outlier was not included in the correlation. Nevertheless, this observation is consistent with the observation that airway closure increases with increasing age [30].

*Comments: Effects of deep exhalation confirm the airway-reopening hypothesis. Breath holding causes time-dependent preferential settling of larger particles in the airways, thereby preventing their exhalation.*

A Hannover research group presented two parallel studies in 2010 [18, 35]. Schwarz et al. [35] measured exhaled particles, flow rates, and tidal volumes online during single breaths in 21 healthy participants aged 21 to 63 years. Spirometry and lung volumes were obtained. Particle concentrations and size distributions were measured online in a temperature-regulated box at 37 °C using a condensation nuclei counter and a laser spectrometer. Six diameter intervals were found ranging from 0.1 to > 5 μm. The protocol involved varying tidal volumes between 20 and 80% of the forced vital capacity. Tidal volumes < 0.7 L were disregarded because response times of the online measuring devices were too slow. One test assessed intra-participant variability through repeated breathing maneuvers after 2 h rest on the same day and during a second visit within 2 months.

The results showed that the difference between exhaled particle concentrations at the smallest and largest tidal volumes could be more than two orders of magnitude. The count median diameter was 0.3 μm and only about 2% of the particles were > 1 μm and none were > 5 μm. With decreasing expiratory flow rates at a given volume, there was a shift toward fewer and smaller particle sizes, in accordance with the increased preferential gravitational settling of larger particles. The number of particles exhaled in a breath seemed to be more influenced by how fast the exhalation started after inhalation was to RV (i.e. a shorter time for sedimentation) than how close to TLC the inhalation finished. Particle emission was positively correlated to age, as previously observed [23]. Increasing the expiratory flow from about 0.2 L/s to 0.8 L/s resulted in an increase in particle emission by a factor of 3, presumably because of a shorter transit time and less deposition of particles before exhalation. Increasing the inspiratory flow from 0.3 L/s to 1.7 L/s showed an insignificant increase in particle emission. High intra-day and inter-day reproducibility within participants was found, with an average correlation coefficient of 0.92, whereas inter-participant variability was about 2 orders of magnitude.

*Comments: The airway reopening hypothesis was challenged by effects of deep exhalation and the hypothesis was strengthened. The observed inter-individual variation was large, but was assessed by correlations.*

The parallel study by Haslbeck et al. [18] investigated particle formation by rupture of surfactant films using computations in a fluid dynamics model. A simplified instrument comprising a biconcave cylinder < 0.5 mm in diameter modeled the small airway structure. A liquid film was applied with uniform thickness in the middle of a cylinder and blocked the cylinder passage. The model described the thinned circular film before rupture and the associated drop formation. The critical thickness of film rupture was 0.2 μm, allowing for computations of film rupture and drop formation as a function of the parameter’s surface tension (0.1–20 dyn/cm), viscosity, and density. The model did not consider the movements of the wall and the drop in pressure across the film. Particle emission was measured in 16 healthy participants in the same way as in the study by Schwarz et al. [35].

The results showed that high surface tension increases the quantity of droplets and slightly reduces droplet sizes. There was no effect of density and almost no effect of viscosity. The count-median diameter was about 0.4 μm irrespective of parameter variation. Thus, surfactant film rupture in simulated small airways produces particles of the same size distribution as during tidal breathing. The results from the human study confirmed the results found by Schwarz et al. [35].

*Comments: The results of the computational model of fluid dynamics simulating small airway opening are consistent with the airway reopening hypothesis. The effect of surface tension was later confirmed* [19]*.*

**Fig. 2:**
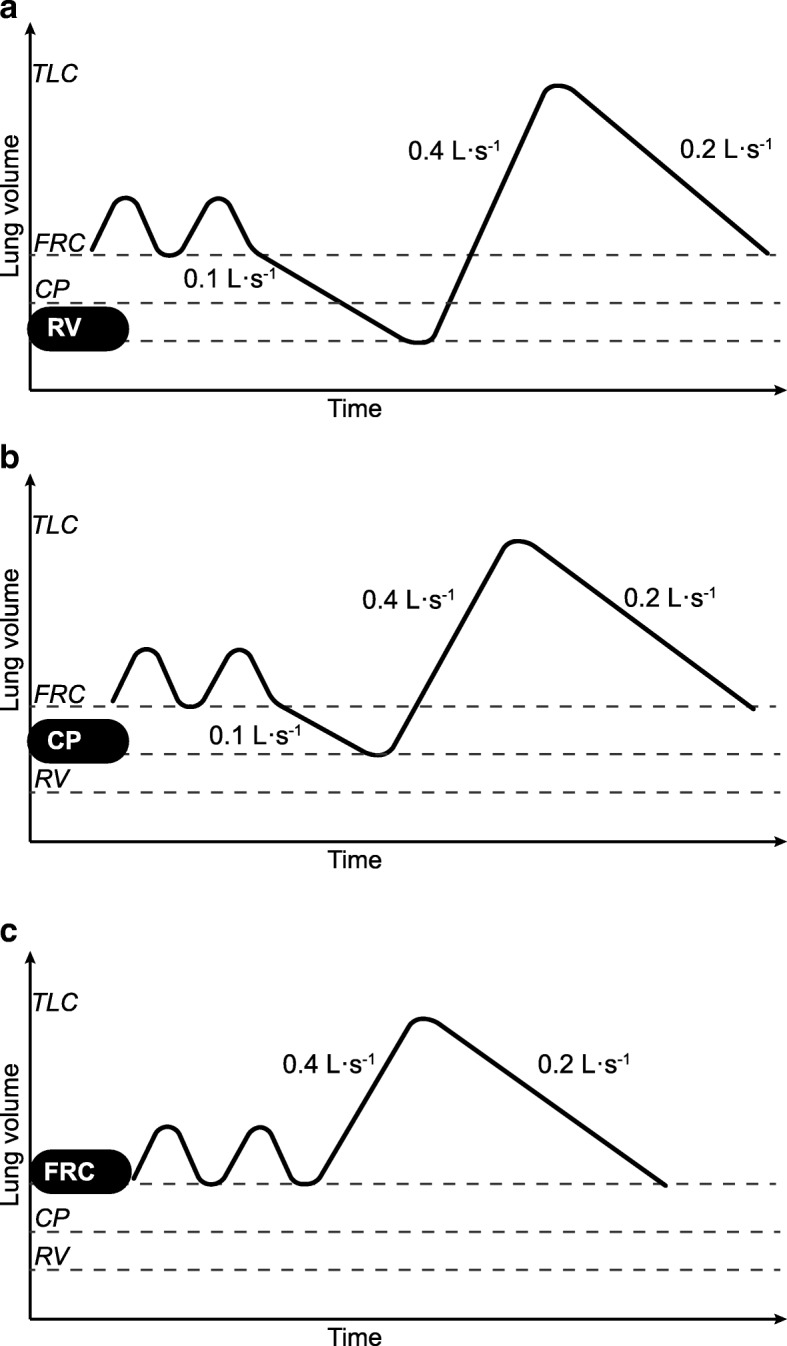
At low specified flow rates the participants exhaled to (**a**) residual volume (RV), (**b**) closing point (CP), i.e., the lung volume at which extensive airway closure begins, or (**c**) normal tidal exhalation to functional residual capacity (FRC). Participants then inhaled to total lung capacity (TLC) and immediately exhaled into the equipment back to FRC. By permission of the author

**Fig. 3:**
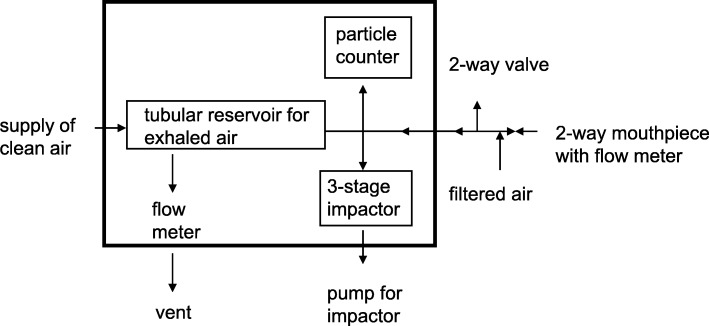
Schematic presentation of the equipment Almstrand et al. [22]. Participants inhale thorough a HEPA filter and exhale into the equipment. The box containing the equipment was maintained at approximately 36 °C. An optical particle sizer and an impactor allowed for counting and sampling of exhaled particles. Exhaled air that was not directly drawn into the impactor and counter was buffered in a reservoir and subsequently drawn into the impactor and counter and replaced by humidified particle-free air. Particles < 4.6 μm were sampled and the size distribution determined. By permission of the author

Almstrand et al. challenged the airway reopening hypothesis by having 10 normal participants performed three strictly controlled breathing maneuvers [22]. Figure 2 illustrates the breathing maneuvers applied to challenge the airway reopening hypothesis. Each maneuver was repeated 10 times. Exhaled particles were counted by an OPC placed inside a box with a thermostat set at 36 °C, drawing a continuous sample from a cylindrical reservoir of 3.4 L capacity inside the box, as described previously in detail [8]. Figure 3 shows a schematic illustration of the equipment counting and sampling exhaled particles. The OPC and sizer determined particles in the range of 0.3 to > 2 μm in diameter subdivided into eight size intervals. Samples from the reservoir of exhaled air were taken until the count rate was close to zero after each maneuver. Then the maneuver was repeated. Count rates combined with the simultaneous flow rates allowed the calculation of the concentration of particles in the exhaled air (n/L).

Average results showed that exhaling to RV produced 8500 n/L, exhaling to closing point (CP) 2500 n/L, and exhaling to functional residual capacity (FRC) 1300 n/L. Particle concentration following the RV maneuver is the sum of the particles generated during inspiration from RV to CP, from CP to FRC, and from FRC to total lung capacity (TLC). Separating the concentrations generated during each of these inspired volume intervals and allowing for the various magnitudes of these intervals showed that the RV-CP interval generated about 85%, the CP-FRC interval about 12%, and the FRC-TLC interval about 3% of the total amount of exhaled particles. The size distributions were not substantially affected by the various maneuvers and the maximum concentration was in the size interval 0.3 to 0.4 μm and there were very few particles above 1 μm.

*Comments: The airway reopening hypothesis is further strengthened. The breathing maneuvers described show that lung volumes where airway closure (and reopening) prevails generate the vast majority of the exhaled particles.*

Holmgren et al. [36] measured the size distribution between 0.01 and 2 μm in 16 healthy participants using an optical particle sizer (OPS) and a scanning mobility particle sizer (SMPS) system. The experimental system was essentially located in a walk-in climate chamber set at 35 °C. The participants sat outside and the OPC measurements were corrected to better represent the actual physical dimensions of exhaled aqueous droplets after recalculation into 15 size intervals from 0.41 to > 33.1 μm. The SMPS system measured particles between 0.01 and 0.43 μm. A 30 L sampling bag collected the exhaled air. Participants performed two breathing maneuvers: (1) normal tidal breathing where the inspirations were of particle-free air and (2) a slow expiration to RV followed by a full inspiration of particle-free air followed by a measured exhalation. Participants exhaled to the sampling bag capacity as the sampling instruments consumed the air in the bag. The breathing maneuvers were repeated twice. The results revealed that during normal tidal breathing the geometric mean particle size was 0.07 μm. During the RV breathing maneuver the particle distribution was mainly between 0.2 and 0.5 μm. There was no correlation between particle emission from tidal breathing and from RV breathing.

*Comments: Tidal breathing emits a mode of extremely small particles, whose mass is negligible. The widely different size distributions resulting from the two breathing maneuvers and the lack of correlation between them suggest different sites of origin.*

Johnson et al. [37] extended previous work [21, 23] and integrated results from the APS assessments of particles with diameters mainly from 0.7 to 20 μm and a droplet deposition analysis (DDA) covering diameter > 20 μm thus spanning a wide range of particle sizes. Their equipment was essentially the wind tunnel set-up described previously [21]. Fifteen healthy participants < 35 years of age participated in the APS studies. Eight were included in the DDA after an oral rinse containing a food dye. As the number of exhaled large droplets was very low, participants had to cough 50 times to produce an adequate number of droplets of each size. The APS counts were corrected for evaporative and dilution effects. Combining DDA results and results from the APS after transformation onto a common scale produced a composite size distribution. Only average results were presented from all individuals due to very large inter- and intra-individual variation. The analysis applied the mixture model assuming log normal distributions.

Results indicated three modes of particle size distributions:

1. Normal and deep tidal breathing resulted in the first mode with a count median diameter of 0.8 μm interpreted to have been caused by the airway reopening mechanism.
2. Speaking, unmodulated vocalization, and coughing resulted in the second mode with a count median diameter of about 1 μm interpreted to have been caused by vocal cord vibrations and aerosolization in the laryngeal region.
3. Speaking and coughing also resulted the third mode with DDA stain dots with a count median diameter of about 200 μm interpreted to have been produced in the presence of saliva, i.e., between the epiglottis and the lips.

*Comments: In addition to previously identified size modes, there was a mode of large particles. This mode relates to particle generation in the upper respiratory tract, including the oral cavity.*

Holmgren et al. [38] extended the study by Johnson and Morawska [23] on breath holding by studying it at both low and high lung volumes. The equipment was the same as that of Almstrand et al. [22]. Ten participants held their breaths at TLC or RV. A breath-hold of 5 s at TLC reduced the exhaled concentrations by as much as 43% and more with longer breath holding, almost certainly due to the settling of particles in the airways. A breath-hold at RV, however, increased the number of exhaled particles: 5 s caused an increase of 63% over no breath-hold, and a 10-s breath-hold caused an increase of 110%. This result was interpreted as an effect of time on airway closure: the number of airways that close at RV increases with time.

*Comments: One ought to consider the time-dependent generation of particles in small airways and the deposition of particles in the alveoli and airways when interpreting results or designing breathing maneuvers.*

Several studies have found that the amount of exhaled particle varies between individuals by orders of magnitude [15, 21, 22, 35]. Bake et al. [16] studied the inter-individual variability of exhaled particle emission in 126 healthy middle aged participants following a standardized breathing maneuver (expiration to RV, breath-holding for 3 sec, full inspiration to TLC, and immediate full expiration into the equipment for measurement of the exhaled particles). The equipment was the same used in the study by Almstrand et al. [22]. Exhaled particles were distributed log-normally with no sign of two superimposed distributions. The inter-individual variation of particle emission was within one order of magnitude, less than previously reported, presumably because of the standardized breathing pattern. Considering age, weight, and spirometry variables reduced the variability further. These predictors explained 28 to 29% of the inter-individual variation, but the remaining variation is still large.

*Comments: Unexplained large inter-subject variability remains.*

**Fig. 4:**
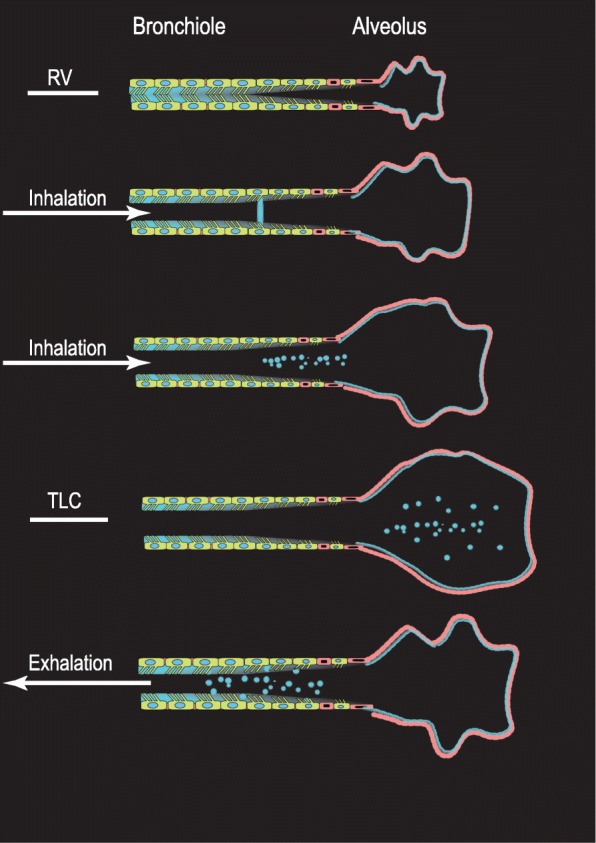
Schematic illustration of the airway reopening concept. When airways close, opposing airway walls get in contact creating a plug of respiratory tract lining fluid. As the airway walls distend during inspiration, forming a meniscus that finally breaks and generate particles. By permission of the author

Figure 4 Illustrates the airway reopening mechanism. As the airways widen during inspiration, closed airways reopen, producing small particles as the plug of RTLF ruptures.

### Chemical evidence of origin

Chemical analysis of exhaled particles can shed light on the origin and mechanisms involved in formation process. Papineni and Rosenthal [14] used electron microscopy and an X-ray dispersive technique for the elemental analysis of droplet residues. They found significant content of potassium, calcium, and chlorine, all abundant in body fluids. Almstrand et al. [8] analyzed impacted particles using the surface-active mass spectrometry, TOF-SIMS. The analysis showed strong signals from phospholipids in all samples from four healthy participants. The identified phospholipid compound groups, such as phosphatidylcholine, phophatidylglycerol, and phosphatidylinositol, are known constituents of surfactants from analyses of bronchoalveolar lavage.

**Fig. 5:**
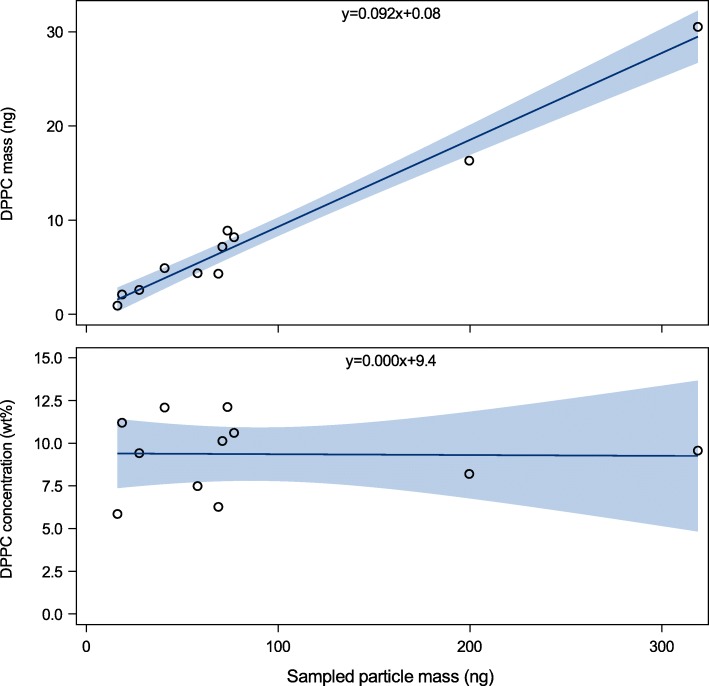
DPPC (1,2-dipalmitoyl-sn-glycero-3-phosphocholine) mass and exhaled sampled particle mass as determined in samples from eleven individuals that performed ten exhalations using the airway reopening maneuver. Note the linear association through the origin between the collected particle mass and collected DPPC mass. The DPPC weight percent concentration (wt%) is shown for each sample in the lower panel. By permission of the author

Several publications have confirmed that exhaled particles contain phospholipids and proteins similarly to surfactant [7–10, 40] thus supporting the origin from RTLF and the airway reopening hypothesis. Of special interest are the phospholipid dipalmitoylphosphatidylcholine (DPPC), a major component of surfactant and known to be produced by alveolar type II cells, and the surfactant protein A (SP-A). Larsson et al. have shown a linear relationship through the origin between analyzed mass of collected albumin, SP-A, DPPC and palmitoyl-oleoyl-phosphatidylcholine (POPC) and the mass of collected particles [10, 41]. Estimations of the mass of the collected particles are based on an optical particle counter providing eight size intervals within the size range about 0.4–4.6 μm in diameter (Grimm Aerosol, Ainring, Germany). Assuming unit density (1000 kg m^− 3^) and spherical particles, the mass of the collected particles can be estimated and the particle concentration of chemical compounds may be given as weight percent (wt%). The relationship between DPPC mass and mass of exhaled particles is illustrated in Fig. 5.

*Comments: This procedure facilitate a normalization of the results and provides an estimate of the concentration in the RTLF of small airways.*

Larsson et al. studied exhaled particles generated by the airway reopening mechanism and during high expiratory flows [10]. Studies were conducted on exhaled particle amounts, particle size distribution and particle content of DPPC. It was expected that the concentration of DPPC would decline with distance from the alveoli due to degradation, dilution and uptake. Exhaled particles’ sizes and concentrations were measured by the instrument previously described by Almstrand et al. [8]. A triple quadrupole mass spectrometer quantified the extracted DPPC content of exhaled particles. Eleven participants aged 28 to 75 years participated and performed four specific breathing activities in a randomized order.

- The *FRC reference maneuver*: tidal breathing, inspiration to TLC, and exhalation into the measuring equipment. These results served as baseline values of particle formation.
- The *forced exhalation maneuver* differed from the FRC reference maneuver only by the high expiratory flow rate, intended to result in exhaled particles produced during the exhalation.
- A *cough maneuver* was included, known to generate a high amount of particles.
- The *airway reopening maneuver*: expiration to RV before inspiration to TLC and exhalation into the equipment. This breathing maneuver induced high amounts of particles generated by the airway reopening mechanism.

The results showed that the *forced exhalation maneuver* and the *cough maneuver* increased the mass of exhaled particles/L exhaled air, compared to the *FRC reference maneuver* by 150 and 640% respectively*.* However, exhaled DPPC mass did not increase over the *FRC reference maneuver*. The *airway reopening maneuver*, however, resulted in a 470% increase of the mass of exhaled particles/L exhaled air over the *FRC reference maneuver* and there was a proportional increase in DPPC. Furthermore, the concentration of DPPC in the particles was similar for the *airway reopening maneuver* and the *FRC reference maneuver.* Thus, forced expirations induce particle formation in the range of 0.4–4.6 μm and these particles contain very little alveolar surfactant. The mass fraction of large particles ranging from 3.0 to 4.6 μm increased after a forced exhalation over the *airway reopening maneuver*, presumably because large particles have a higher probability of being exhaled when formed in central or upper airways during forced exhalations due to short transit times.

*Comments: Central airways as well as small airways generate exhaled particles but with different compositions.*

Ljungkvist et al. [44] measured concentration of methadone in exhaled particles of 13 participants receiving methadone maintenance treatment. The PExA impaction method (PExA®) measuring particle sizes from 0.4 μm to 4.6 μm was compared to an electret filtration method collecting particles of any sizes during tidal breathing [42, 43]. All samples by the PExA method during the RV breathing pattern contained methadone. Thus, the methadone distribution includes RTLF of small airways. Interestingly, the filtration device collects substantially more methadone than the impaction instrument, almost certainly because the filtration device collects larger particles from upper airways and/or oral fluid.

*Comments: It is confirmed that exhaled particles during tidal breathing include relatively large particles that dominates the exhaled mass and that is not associated to airway reopening.*

### Particles in exhaled breath – A potential biomarker of small airway disease?

Chemical analysis of exhaled particles provides huge possibilities to explore the biomarkers of small airway diseases, and we are probably just beginning to utilize this new biological matrix to its full extent.

Particle emission among patients with COPD appears unclear [39, 45]. Schwarz et al. [39] reported that there were no differences in particle number concentrations between healthy nonsmokers (*n* = 16) and COPD patients (*n* = 28). However, the COPD patients presented in their Fig. 2 clearly emit less particles than the healthy non-smokers do. Lärstad et al. [45] reported substantially reduced particle emission in COPD patients (*n* = 13) compared with healthy participants (*n* = 12). Despite some ambiguity, we consider the results to indicate that COPD patients exhale fewer particles than healthy participants. One reason may be that hyperinflation in the COPD patients prevents their ability to expire to low lung volumes. When healthy participants exhale to CP rather than to RV, their particle emissions were about one third of that at RV [22]. Another reason may be that terminal bronchioles are destroyed in COPD, [46] resulting in fewer small airways to close and open. Furthermore, available airways may be injured due to the mechanical stress of cyclic closing and opening [3, 33], possibly affecting particle composition and production.

Lärstad et al. [45] studied SP-A and albumin concentrations using PEx. SP-A is involved in many biological processes in the lung periphery associated with inflammation [47] and is an interesting potential biomarker in particles from small airways. Albumin concentrations may, among other things, be an indicator of plasma leaking into the airways [48]. SP-A was determined by enzyme-linked immunosorbent assay and the results showed that among COPD patients particle concentrations of SP-A (wt%) were lower than in healthy controls whereas albumin levels were similar.

*Comments: Small airways are indeed involved in COPD, as shown by the comprehensive studies by Hogg* et al [49–51] *and several mechanisms of particle production may be operative.*

Patients with asthma (*n* = 10) before and after metacholine challenge were studied by Schwarz et al. [39]. Particle emissions were found to be no different between healthy non-smoking participants (*n* = 16), and metacholine challenge did not affect particle emission despite significant bronchial obstruction. Larsson et al./ [41] studied particle emission and particle content of SP-A and albumin in birch-pollen allergic participants with asthma (*n* = 13) and healthy controls (*n* = 13). The exhaled particle emissions decreased during pollen season among asthmatic participants but was unchanged among controls. SP-A (wt%) and albumin (wt%) were no different between asthmatic participants and controls, and there were no effects of pollen season. Thus, results of particle emissions from asthmatic participants are inconsistent. At the European Respiratory Society International Congress in Milan 2017, Östling et al. presented the proteomics (SomaLogic, Inc., CO, USA) of small airway RTLF [11]. PEx was collected during the RV breathing pattern by the PExA instrument in 20 participants with asthma and 10 healthy controls. Over 200 different proteins were detected. Many proteins were different in asthmatic patients to those in controls and there was a striking age dependence in many proteins. The obtained protein profiles from the small airways suggest that the method captures pathobiologically relevant proteins and that a specific profile indicates an asthma sub-phenotype.

*Comments: Proteomics from small airways offers an exciting potential to obtain a fingerprint from small airways.*

Rhinovirus infected participants (*n* = 16) were studied by Fabian et al. [24]. Participants were instructed to breathe normally for 20 min into the equipment. Exhaled particles were collected on gelatin filters for rhinovirus quantification. Results were negative, indicating either that the amount of virus was below the limit of detection or that the virus was not present in the collected particles. The study included three healthy volunteers to study the effect of coughing, swallowing, tidal breathing and breathing to TLC and RV. Exhaled particle concentrations were observed to increase 10 to 70 times when participants exhaled to RV before inhalation to TLC.

*Comments: Results consistent with the airway reopening hypothesis.*

Patients with bronchiolitis obliterans syndrome (BOS) after lung transplants showed lower SP-A particle concentrations than the BOS free group of lung transplants [52].

Analysis by TOF-SIMS has shown that phospholipids of smokers are more protonated and sodiated than those of non-smokers [53]. As the particles were produced by the airway opening mechanism, the results indicate the effects of smoking on the RTLF of small airways. Smokers also were studied by Schwarz et al. [39] but no effects on particle emission were found.

## Conclusions

Various mechanisms generate particles appearing in exhaled breath. The sites of origin differ, depending on the breathing maneuver applied. The process of reopening small airways is one scientifically substantiated particle generation mechanism. Analyzing the content of exhaled particles as generated by the airway reopening mechanism, offers an exciting noninvasive way to obtain samples of RTLF from small airways. Results from a few early and small clinical studies on COPD, asthma and BOS indicate associations with altered particle formation and particle composition. In the future, analysis of exhaled particles may provide a “fingerprint” of small airways revealing important biomarkers.

## Additional file

Additional file 1: Technical and methodological considerations. (DOCX 21.4 kb)
