# Supplementary material for: Exhaled particles and small airways
Source: Respir Res. 2019 Jan 11;20:8. doi: 10.1186/s12931-019-0970-9 (PMC6330423; doi:10.1186/s12931-019-0970-9)
Supplement: Supplementary file 1 — Technical and methodological considerations. (DOCX 21.4 kb) [file 12931_2019_970_MOESM1_ESM.docx]

SUPPLEMENT 1 Technical and methodological considerations

The supplement gives some background to the various measurement techniques employed in the work cited in the present review. The basic principle behind a measurement device, some common technical limitations and the abbreviations often used in the literature are given. However, the actual measurement setup used in an investigation is beyond the scope of this Supplement.

*Airborne particles*

An aerosol is a collection of condensed matter, i.e. solid or liquid particles, suspended in air. The particles should have a “life time” and not immediately deposit by gravitation, something that limits the physical size upwards to a few tenths of a millimeter (mm). An important characteristic of an aerosol is its particle size distribution. Such a distribution may be based on measured number or mass versus particle size. If the number or mass data is plotted with a linear x-axis it is often seen that there is a "tail" towards large particle sizes. If, however, a logarithmic x-axis is used, the plot becomes more symmetric and looks similar to a normal distribution. There is no sound theoretical reason why aerosol data should behave in this way but the lognormal distribution is often a good approximation, especially if the aerosol has a single source. Therefore, the parameters defining the distribution with geometric mean diameter D_g,_, geometric standard deviation sigma_g_ and total number or mass concentration is often used to describe an aerosol.

Particles generated in situ and then exhaled, are expected to be liquid spheres and the particle volume can be derived from the sphere diameter (D). A twofold increase in diameter thus results in an eightfold increase in volume (and mass).

Aqueous droplets give off or pick up water vapor to establish an equilibrium with the surrounding air. It follows that droplet size depends on particle composition, air temperature and humidity. Equilibration is a rapid process (< 1 s) for small droplets. A confounding factor may be the presence of a surfactant layer covering the droplet surface, possibly slowing down evaporation/condensation.

It is difficult to directly determine size of small droplets (D< 10 µm) floating in air. In practice, the size is usually derived indirectly from a measured property that depends on particle size but is more easily obtained. The various sizing methods employed in the cited work are described below.

*Direct methods*

Droplet Deposition Analysis (DDA), sometimes assisted by optical or electron microscopy uses a surface that retains traces of deposited droplets. The size of a droplet trace depends on the original droplet and may be related by a scale factor that has to be determined by calibration. If a trace is measured by a reticle then the original droplet size may thus be calculated. Colored droplets or a thin surface layer of powder have been used to obtain measurable traces. Experimental difficulties e.g. with identifying true traces often limit the method to droplets larger than about 10 µm.

*Light scattering*

An illuminated particle will scatter light away from the incoming light path. This light may be detected and its intensity measured. Scattering is the functional basis for Optical Particle Counters (OPC) and Optical Particle Sizers (OPS). The process is known as Mie scattering and is a special case of Maxwell's theory of electromagnetic fields that occurs when the particle dimension is similar to or larger than the wavelength of the light. Light scattered from different parts of the spherical particle will interfere and give an intensity pattern that depends on wavelength, scattering angle, particle size and its optical properties. The calculation e.g. of scattered intensity is not trivial.

For counting, typically, the aerosol is sent through a narrow orifice so that only one particle at a time is illuminated, giving rise to a pulse of scattered light that may be recorded. The arrangement is often such that aerosol flow and incident light are at right angles while detection of the scattered light is made along the third orthogonal direction. The scattering power of small particles (≈ 0.2 µm) may easily drop by a factor of ten if the diameter is reduced by a factor of two. This limits the sizes detectable to between 0.1 and 0.3 µm. Counting pulses and knowing the flow rate give the number concentration.

A common arrangement to measure very small particles ( d<0.1 is the Condensation Particle Counter (CPC) or (synonymous) Condensation Nuclei Counter (CNC). A CPC works by first saturating the aerosol with a volatile substance e.g. butanol or water at a slightly increased temperature. Then the aerosol is passed through a cooled section, lowering the temperature and supersaturating the gas, forcing the volatile substance to condense on the particles, thereby growing them to a size detectable with light scattering. The smallest detectable particle size is dependent on the degree of supersaturation achieved. However, the original size distribution is lost by this method.

The intensity of scattered light in a given direction is a measure of particle size but also depends on light wavelength, particle light absorption and its refractive index. This is exploited in the Optical Particle Sizer (OPS). In this type of instrument, not only are the scattered light pulses counted but also their intensity measured. There is no simple way to calculate size from first principles. Instead, an OPS should preferably be calibrated using particles of known size and refractive index. The low scattering power of small particles limits the useful size range downwards to 0.1-0.2 µm. In current literature, mentioning "optical particle counter" (OPC) together with "size bin" or size bin limits is a strong indication that the instrument is in fact an OPS.

By measuring the angular scattering pattern of monochromatic light from an aerosol and applying Mie theory it appears possible to deconvolute the pattern to give the original aerosol size distribution. This technique is known as "laser diffraction" (LD) and devices capable of giving a complete size distribution from 0.1 to 600 µm are available. The low concentrations often encountered for exhaled aerosols may however in practice limit the usefulness.

Yet another laser based measurement system is the Interferometric Mie Imaging technique (IMI). Here a thin sheath of laser light is passed by the aerosol particles. The light is scattered and refracted by a particle on its passage through the light sheath and a specific camera (CCD) records the interference pattern between the scattered and refracted light. By analyzing the CCD images and applying Mie theory it is possible to obtain a size distribution between 2 and 2000 µm. The technique allows analysis of particle size in situ, close to the source without the need to use tubes or ducts.

*Particle dynamics*

The dynamic behavior of a particle may reveal its size. According to Stokes's law, the force needed to move a spherical object of diameter D at velocity V is linearly dependent on these quantities. Stokes's law is subject to the requirement of low Reynolds number. This is often fulfilled at the low velocities and small particle size of aerosol particles.

If a particle, smaller than ≈ 20 µm, provided with an electrical charge, and exposed to an electrical field, it will increase its velocity until the drag force from the surrounding air balances the electric force. The particle velocity together with the electrical field strength yields the diameter of the sphere. This is implemented in the Differential Mobility Analyzer (DMA) where a narrow size band may be selected from an aerosol by setting the electrical field. A DMA followed by a particle counter, comprise a Scanning Mobility Particle Sizer (SMPS) system. Such a system may be used to determine particle sizes from around 0.005 to 1 µm. As the name implies, the instrument scans over a size range making it slow and unsuitable to follow transient processes.

An Aerodynamic Particle Sizer (APS) sizes particles according to their inertia that depends on mass i.e. on size. The aerosol is sent through a nozzle where airflow and particles are accelerated. The particle acceleration and final velocity depends on its inertia. Particle velocity is measured by timing the passage of a particle between two laser beams separated by a small distance. A passing particle gives two pulses of scattered light and the time delay between the pulses depends on velocity and thus on the inertia of the particle. This information allows calculation of the particle aerodynamic diameter. The aerodynamic diameter D_a_ of a particle is the diameter of a sphere with unit density that has the same settling velocity as the particle thereby reflecting its aerodynamic behavior. The physical size D of a spherical particle may be calculated from D_a_ and its density ρ as D = D_a_ *ρ^-½^. Often, an APS also records the intensity of the scattered light, allowing operation also as an OPS. The size range of the APS covers diameters between approximately 0.5 and 20 µm.

Yet another device based on particle inertia is the "impactor". It allows for collection of a size discriminated sample from an aerosol e.g. for weighing or for chemical analysis. A vacuum pump draws the aerosol through one or several equally sized orifices where the gas velocity is increased since the cross section is reduced but the volume is constant. Downstream of the orifices and arranged perpendicular to the gas stream(s), is an impaction plate causing a 90° deflection of the gas flow. For a given air velocity, particles with a small diameter have a lower inertia and can alter direction and follow the air stream around the impaction plate while larger particles cannot and impact on the plate and deposit. The inertia property is used for designing impactors to collect particles above a certain size. Their cutoff or D_50_ number often describes impactors. This number describes the aerodynamic particle diameter where the collection efficiency is 50%. Thus, particles larger than the d_50_ diameter have a higher than 50% probability to be collected whereas smaller particles have a lower than 50% probability to be collected. By stacking several orifice/impaction plate units with sequentially decreasing total cross-section of nozzles, resulting in increasing orifice gas velocity, it is possible to separate particles into "size bins". Such a device is known as a cascade impactor and the sharper the cutoff curve for each stage is the smaller the overlap of particle sizes between the bins becomes.
